# Revisiting Transverse Myelitis: Moving Toward a New Nomenclature

**DOI:** 10.3389/fneur.2020.519468

**Published:** 2020-09-23

**Authors:** Kyle M. Blackburn, Benjamin M. Greenberg

**Affiliations:** ^1^Department of Neurology, University of Texas Southwestern Medical Center, Dallas, TX, United States; ^2^Department of Pediatrics, University of Texas Southwestern Medical Center, Dallas, TX, United States

**Keywords:** transverse myelitis (TM), myelopathy, myelitis, inflammatory myelopathy, spinal cord demyelinating lesions

## Introduction

Since its first use in case reports in the early twentieth century, the term transverse myelitis has become the preferred label for immune-mediated myelopathies. In 2002, diagnostic criteria created by an expert consensus defined transverse myelitis as a syndrome, divided into disease-associated transverse myelitis (i.e., myelitis attributed to a recognized disorder such as multiple sclerosis), and “idiopathic transverse myelitis” where no underlying cause is identified after comprehensive evaluation (1). In the years following publication of these criteria, neuroimaging research and biomarker discovery have provided important insights into the pathophysiology of many neuroimmune disorders. Accordingly, updates to clinical guidelines and diagnostic algorithms are needed to reflect a modern understanding of inflammatory myelopathies. Here, we discuss issues with the blanketed use of “transverse myelitis” and propose that the term be retired in future classification systems.

### Immune-Mediated Myelopathies Are Radiographically Heterogeneous

While it has been widely propagated that the term transverse myelitis was first used by Dr. Suchett-Kaye in 1948, we find the term in case reports dating back to 1931 (2, 3). Though not explicitly stated in these reports, it is generally felt that the use of “transverse” was meant to reflect involvement of the entire axial plane of the spinal cord. While the significance of these early reports of spinal cord inflammation cannot be understated, it is now apparent that the landscape of immune-mediated myelopathies includes a wide spectrum of presentations with diverse imaging characteristics. The use of a catch-all term like transverse myelitis does not accurately reflect these complexities.

Involvement within the transverse plane is highly variable amongst myelopathies, and several causes have defining imaging characteristics. Multiple sclerosis classically causes a partial myelitis with predilection for the white matter tracts, while other causes may result in a mix of gray and white matter involvement (4). In recent years, outbreaks of a gray-matter centric myelitis associated with enterovirus D68 (EVD68) have resulted in flaccid paralysis in children (5). Such examples highlight the nuance in characterizing myelopathies in the transverse plane.

Furthermore, the extent of involvement within the rostral-caudal dimension also has important implications in myelopathy evaluations. Longitudinally-extensive myelitis (typically defined as greater than 3 vertebral segments long) carries different differential considerations than short-segment myelitis, and can be the hallmark of a recurrent disorder such as neuromyelitis optica spectrum disorder (4). Other spatial characteristics such as subpial involvement in sarcoidosis (6), also carry significant weight in the evaluation of immune-mediated myelopathies.

### “Transverse Myelitis” Does Not Inform on Etiology

The diagnostic approach to acute myelopathies can be challenging given the extensive differential diagnosis. While it is generally understood among clinicians that transverse myelitis implies an inflammatory etiology, the term does not make this distinction clear. Significant advances in the understanding of myelopathies allow for a more refined understanding of etiology, which should be reflected in terminology. Important discoveries, such as antibodies to aquaporin-4 and myelin oligodendrocyte glycoprotein (MOG) are not accounted for in current clinical criteria of transverse myelitis (7). Furthermore, efforts to improve diagnosis of vascular myelopathies provide an opportunity to increase recognition and avoid risks of immunotherapies in certain patients (8). By labeling myelopathies by etiology, clinicians are better able to prognosticate and provide appropriate treatments, and patients will have a better understanding of their overall condition.

### The Term Can Create Barriers in Communication Between Patients and Clinicians

The term transverse myelitis is often applied in two related, yet distinct, scenarios. In one instance, it is used to describe spinal cord disease associated with a neurologic or systemic autoimmune disorder. Patients with disease-associated myelitis frequently require close surveillance and treatment with immunotherapies to prevent new inflammation within the spinal cord or elsewhere. In the second scenario, the term transverse myelitis is used by clinicians as a shortened form of “idiopathic transverse myelitis,” denoting an inflammatory myelopathy of unclear etiology. While neurologists are experienced in navigating transverse myelitis as both a syndrome and a distinct diagnostic entity, patients may not understand this difference when presented with a new diagnosis. Upon researching their disorder, they may grow concerned they have two unique neurological diseases, causing significant confusion and anxiety about their prognosis. Eliminating medical terms with multiple potential meanings from our lexicon serves to improve physician-patient communications and foster a constructive partnership.

## Proposed Framework for a New Nomenclature

**Figure 1:**
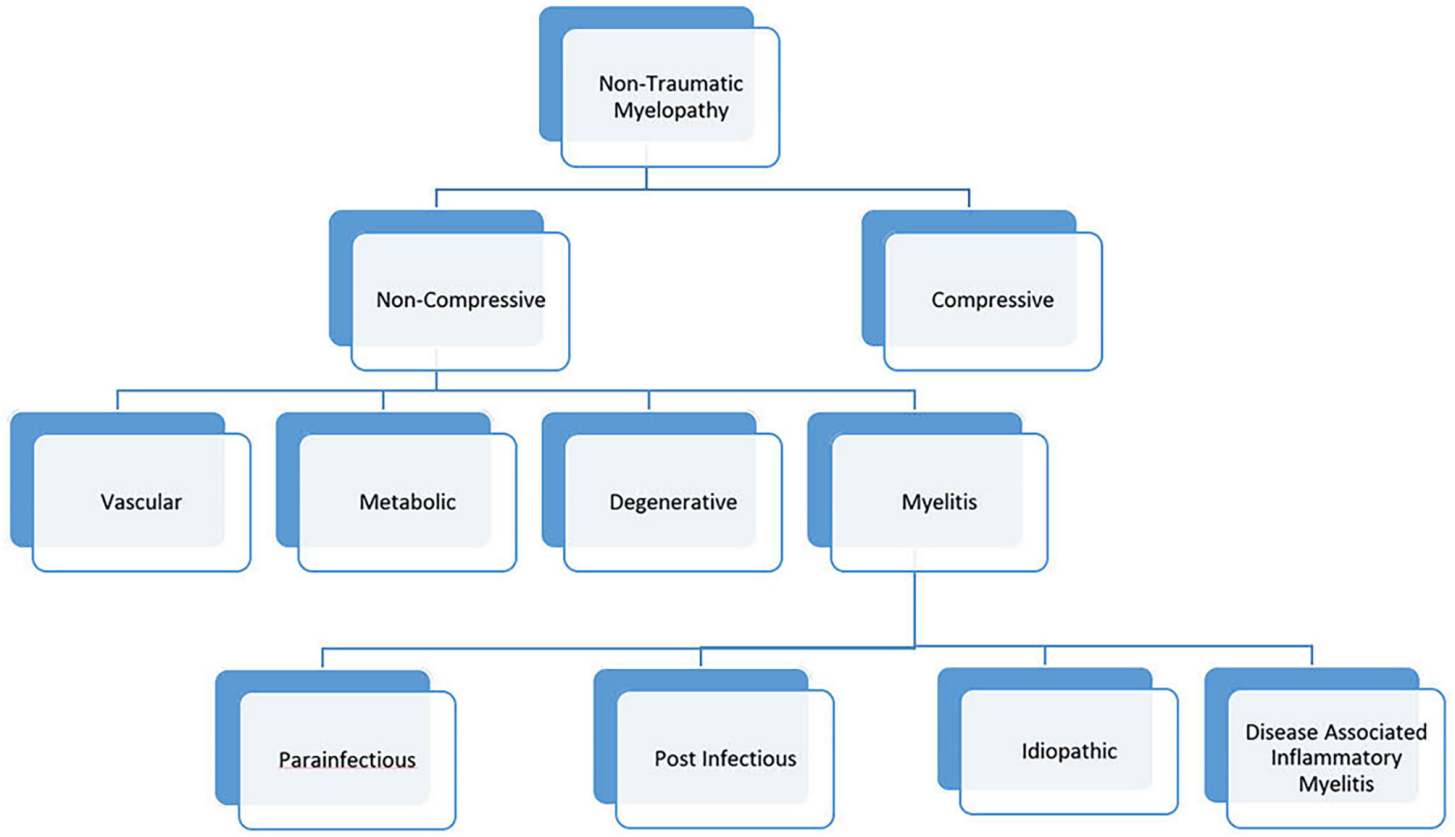
Etiologic classification of myelopathies. In this proposed framework, the term “myelitis” is used to define any clinical presentation of myelopathy with evidence of inflammation on imaging or CSF analysis.

Given significant advances in myelopathy research, a revision to the 2002 working group criteria is needed. We propose a new naming convention for myelopathies, in which “myelitis” is used to describe myelopathies with evidence of inflammation on neuroimaging or cerebrospinal fluid analysis (Figure 1). This category would include both infectious and immune-mediated myelopathies to promote a comprehensive evaluation in myelitis. Cases of myelitis associated with an infectious pathogen could be further divided into para-infectious and post-infectious myelitis. This nomenclature would recognize that a parenchymal spinal cord infection (e.g., EVD68) can illicit an immune response causing damage (parainfectious) vs. a myelitis event caused by a deranged immune system that was triggered by a prior systemic infection (post-infectious). Similar to the 2002 criteria, myelitis associated with a known neuroimmune or systemic autoimmune disorder would be known as disease-associated inflammatory myelitis. After a comprehensive evaluation, myelitis of unknown etiology could be simply labeled “idiopathic myelitis.”

In summary, it is time to retire the term transverse myelitis and overhaul current classification systems to cultivate modern, coherent definitions for myelitis.

## Author Contributions

KB participated in drafting and editing the manuscript. BG formulated the concept, and participated in drafting and editing the manuscript. Both authors contributed to the article and approved the submitted version.

## Conflict of Interest

BG has received grant support from the NIH, PCORI, NMSS, Guthy Jackson Charitable Foundation for NMO, Genentech, Chugai, Medimmune and Medday. He has received consulting fees from Alexion and Novartis. He serves on the advisory board for the Siegel Rare Neuroimmune Association. KB Fellowship was funded by the Siegel Rare Neuroimmune Association.
